# The Effects of Different Substrates in Pond Net Cages on the Succession of Periphyton and the Seedling Protection of Sea Cucumber *Apostichopus japonicus*

**DOI:** 10.3390/biology15020182

**Published:** 2026-01-19

**Authors:** Yanqing Wu, Liming Liu, Rongbin Du, Wengang Xu, Bo Qin, Na Ying, Bianbian Zhang

**Affiliations:** 1 East China Sea Fisheries Research Institute, Chinese Academy of Fishery Sciences, Shanghai 200090, China; wuyanqing0961@163.com (Y.W.); q_balance@foxmail.com (B.Q.); xyz6622@126.com (N.Y.); zhangbianbian@hotmail.com (B.Z.); 2 School of Ocean, Yantai University, Yantai 264005, China; liu_liming71@163.com (L.L.); rbdu62@163.com (R.D.); 3 Innovation Center of Lianyungang, East China Sea Fisheries Research Institute, Lianyungang 222000, China

**Keywords:** aquaculture, survival rate, yield, chlorophyll-a, pheophytin-a, dry weight

## Abstract

With the industry development of sea cucumber *Apostichopus japonicus* aquaculture, the indoor high cost and low survival rate have become serious problems. Therefore, it is necessary to optimize substrate selection for seedling protection in outdoor pond net cages. This study explores the succession of periphyton on the different substrate surface types, including a curvimurate net (CU), nylon mesh (NM), and ground cages (including a ground cage net (CN) and ground cage plate (CP)), and their effects on the seedling protection of sea cucumbers. In addition, we monitored the substrates’ dry weight, chlorophyll-a, and the community composition of substrates, alongside seedling growth, yield, and survival rate. The results show that a total of 7 phyla, 23 genera, and 31 species were detected on the substrates, with diatoms dominating (19 species) and Chlorophyta (4 species) being the main species. The CU had the highest total number of alga species attached, significantly higher than the other substrates in week 13 (*p* < 0.05). In week 9, the diatom density dropped to its lowest point, and, after September, it rose with the decrease in water temperature. In terms of dry weight with and without ash, CP increased rapidly in the early stage, with NM, CU, and CP being significantly higher than CN in week 13 (*p* < 0.05). The chlorophyll-a content showed a decreasing–increasing–decreasing trend, with CU reaching 3.62 ± 0.48 μg/cm^2^ in the 13th week, significantly higher than other substrates (*p* < 0.05). Finally, the *A. japonicus* survival rate and yield in the CU group at week 12 were significantly higher than those in the NM and ground cage groups (*p* < 0.05). At week 17, the average weight, yield, and survival rate in the CU group were still optimal, with the yield 5.76 times that in the initial dosage. These results suggest that the CU has a suitable mesh size, has good permeability, and may stably support sediment, which is conducive to the growth of benthic diatoms. In addition, it can provide sufficient natural feed and a good habitat environment and is the preferred substrate for *A. japonicus* seedling protection in outdoor pond net cages.

## Simple Summary

This study set up net cages with different materials in a sea cucumber *Apostichopus japonicus* aquaculture pond. Experiments were conducted to compare the different types of attachments—curvimurate net (CU), nylon mesh (NM), and ground cage—aiming to understand the occurrence and succession of surface-attached organisms on different types of adhesive substrates and their effects on the cultivation of sea cucumber seedlings. The results indicate that, in the early stage, benthic diatoms dominate, while, in the later stages, blue algae and green algae dominate. However, the net cage with the CU substrate has the highest density, chlorophyll-a content, and number of alga species, resulting in a significantly higher seedling yield and survival rate than the other substrate materials. Therefore, among the different attachments, the CU substrate is the best choice for *A. japonicus* seedling culture.

## 1. Introduction

The sea cucumber, *Apostichopus japonicus*, belongs to Echinodermata, Holothuroidea, Stichopodidae, and *Apostichopus* [1]. Its high nutritional and medicinal value, for which it is known as “sea ginseng”, means it is one of the important economic sea cucumber varieties in China [2]. With the increase in market demand and improvements in seedling and breeding technology, the scale of the sea cucumber industry has rapidly expanded [3], and *A. japonicus* culture has sparked the fifth wave of aquaculture nationwide, following the cultivation of kelp, shrimp, shellfish, and fish, developing into a pillar marine aquaculture industry in northern China. By the end of 2024, the aquaculture production of *A. japonicus* in China had reached 292,045 tons [4].

In recent years, with the continuous expansion of the *A. japonicus* cultivation scale, the market demand for high-quality seedlings has been increasing. The traditional indoor intermediate cultivation method, with high production costs, long cycles, and low survival rates, directly released young *A. japonicus* into ponds for cultivation and is now unable to meet the needs of the *A. japonicus* cultivation industry [5]. With the gradually declining price of *A. japonicus* seedlings in recent years, a low-cost and effective intermediate cultivation method, outdoor pond net cage seedling preservation, has become popular [6].

*A. japonicus* generally inhabits substrates rich in benthic organisms in natural waters [2], which not only provide them with shelter but also natural food [7,8]. Artificial substrates, such as attachment facilities in *A. japonicus* farming, play an important role in their growth process [9]. In *A. japonicus* outdoor pond seedling preservation, net cages are usually adopted to preserve and facilitate the centralized capture of seedlings [10]. To date, there have been some reports on the effects of artificial substrates on the growth, survival [11,12], and trends [13] of *A. japonicus* during cultivation. However, there is relatively little research on the development process, effects, or effectiveness in protecting the *A. japonicus* seedlings of artificial substrates in pond cages. To explore the optimal seedling preservation mode that may improve the seedling quality, reduce costs, and increase efficiency during *A. japonicus* cultivation, it is necessary to conduct research on the succession and effects on seedling preservation of different substrates in pond cages [14].

Recently, studies on *A. japonicus* have focused on bioactive compounds and functional properties [15], pharmacological potential [16], metabolites [17], culture [18], and trade [19]. However, little information has been reported on the effects of different substrates on the succession of the periphyton and *A. japonicus* seedling protection. This study compared the dry weight, chlorophyll-a levels, and types and quantities of attached organisms on the surfaces of three types of substrates (four types of attachment substrate materials in total), a curvimurate net (CU), nylon mesh (NM), and ground cages (including a ground cage net (CN) and ground cage plate (CP)), during the process of pond net cage seedling preservation. The study aimed to understand the succession and changes in attached organisms in the pond and clarify the changes in the number of benthic diatoms on the surfaces of attachment substrates during seedling preservation, as well as the growth rate, survival rate, and yield changes in *A. japonicus* seedlings when placed in different attachment substrate net cages. The results may provide a theoretical basis for the selection of attachment substrates for outdoor pond net cage *A. japonicus* seedling preservation.

## 2. Materials and Methods

### 2.1. Experimental Pond and Net Cage

The experimental pond was located at the *A. japonicus* breeding farm of Shandong Oriental Marine Technology Co., Ltd. in Laizhou City, Shandong Province, China, and was conducted from 28 June to 3 November. The pond covered about 20,000 m^2^, with a depth of 1.17–2.0 m, sandy and muddy bottom, complete drainage system, and fresh and pollution-free water. The upper part of the pond was covered with a black sunshade net to lower the water temperature during summer. During the experiment, the water temperature was measured at 08:00 every morning. The salinity, dissolved oxygen, and pH was measured using the water quality analyzer HACH ^®^ TM156 (HACH, Danaher Corporation, Loveland, CO, USA). A 2.5 L organic glass water sampler was used to mix the water from the upper (depth 30 cm), middle (depth 90 cm), and lower (depth 150 cm) ponds separately, and then 1–2 L of the mixed water was taken for measurement. The indicators were measured three times at each sampling. The water temperature changed from 11.2 to 29.9 °C, pH from 7.46 to 8.28, the dissolved oxygen from 7.72 to 11.92 mg/L, and the salinity from 25.51 to 32.48.

**Figure 1:**
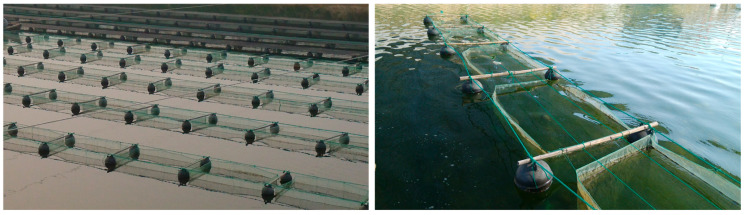
Appearance of net cages in the pond.

The size of the net cages was 2 m × 1 m × 1 m, with a polyethylene mesh at the bottom for attaching detached seedlings. The net cage frames were made of spherical floats, thick nylon ropes, and bamboo poles, with all sides and bottom surfaces made of polyethylene mesh save for an open mouth. The net cages were covered with a black sunshade net about 20 cm above the surface to prevent high water temperature in the summer. The net cages were equipped with a curvimurate net attachment, base size 10 m × 1 m, and a 14 mesh. The thin nylon ropes and stones were alternately tied to the net cage frame every 50 cm, with the stones placed inside the net cage and the net pieces suspended in a wave shape. Each net cage was placed with one curvimurate net (Figure 1).

### 2.2. Specifications and Settings of Substrates

**Figure 2:**
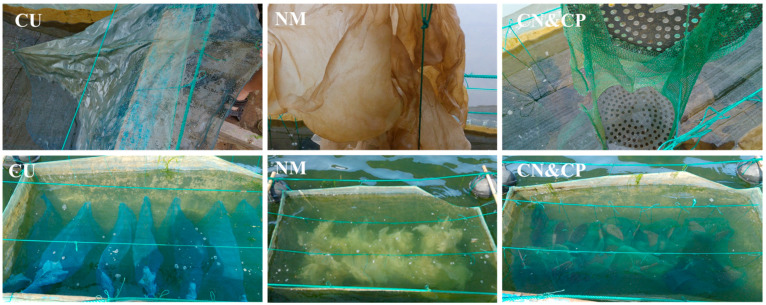
Substrates in net cage. CU: curvimurate net; NM: nylon mesh; CN&CP: cage net and cage plate.

The different types of attachment substrates were selected for the experiment: CU, NM, and CN&CP (net cage including a ground cage net (CN) and ground cage plate (CP)) (Figure 2). The nylon mesh used was 50 cm × 35 cm 20 mesh, used as an attachment base for indoor seedling cultivation and connected in series with ropes, with eight pieces per string and hanging stones below, with each net cage receiving seven strings. For the final substrates, a 7.5 m long ground cage net and 15 ground cage boards (perforated hard plastic boards) were placed in each net cage, and the boards were hung in the net cage with thin ropes 25 cm apart.

### 2.3. Experimental Design

**Figure 3:**
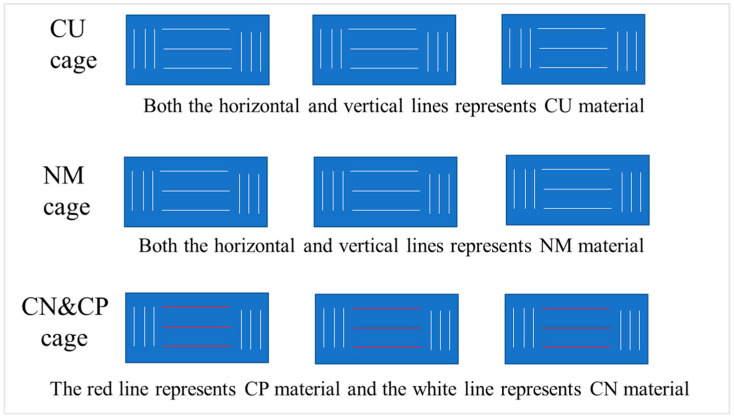
The design of net cage materials with CU, NM, and CN&CP in this experiment.

The CU, NM, and CN&CP substrates were disinfected and then placed in the separate net cages with equal surface areas and three parallel attachments set for each substrate type within the independent net cages. After they were placed, samples were taken every 1–2 weeks. CN&CPs were set and placed in the same cage, while they were sampled and measured separately (Figure 3).

Nine samples of each substrate type were taken from each net cage to determine the dry weight, chlorophyll-a, pheophytin-a, and composition of the organisms on the surface, and three replicates were set for each indicator. Furthermore, the attachment and distribution of *A. japonicus* seedlings were observed, and the average seedling weight, yield, and survival rate were measured at week 12 and week 17.

### 2.4. Seed Breeding and Seedling Conservation Management

Seedling breeding was set up at 0.10 ± 0.09 g/ind, with a total of 4.5 kg of seedlings evenly distributed across the nine net cages for an average stocking density of about 2700 ind/m^3^. They were placed in the CU cage, NM cage, and CN&CP cage, respectively, and were fed daily at 18:00, with the feeding amount calculated from 8.0% to 23.7% of body weight, referenced from Seo et al. [20]. They were fed with specialized artificial bait for young *A. japonicus*, mixed with *Sargassum thunbergii*, *spirulina*, sargasso weed, spirulina, yeast, kelp powder, and sea mud. The amount in each net cage remained the same, and 10% to 20% of the pond water was changed every day.

### 2.5. Sample Determination Method

#### 2.5.1. Detection of Susbtrate Dry Weight and Ash-Free Dry Weight

During each sampling, different pieces of CU, NM, and CN&CP were sampled to measure their length and width. A clean brush was used to scrape off surface attachments, and these were placed in a culture dish. We rinsed the substrate surfaces with particle-free seawater and then filtered them through a 0.45 μm pore size glass fiber microporous filter membrane that had been pre-burned at 450 °C for 6 h and weighed. The resulting samples were then dried in an electric constant-temperature oven at 60 °C to a constant weight to calculate the dry weight (DW), before being burned in a 450 °C muffle furnace for 6 h and weighed again to calculate the ash-free dry weight (AFDW).

#### 2.5.2. Determination of Chlorophyll-a and Pheophytin-a Contents in Substrates

Based on the above method, we brushed the substrate surface attachments, filtered them with a 0.45 μm pore size glass fiber microporous membrane, and extracted them with 90% acetone at 4 °C in a refrigerator for 18–24 h. We measured the optical density at 665 nm and 750 nm with a spectrophotometer, added 1 drop of hydrochloric acid solution to the colorimetric dish, and measured the optical density values at 750 nm and 665 nm again within 5–10 min to calculate the concentrations of chlorophyll-a and pheophytin-a [21].

#### 2.5.3. Analysis of Attached Biological Communities

During sampling, single pieces of approximately 5 cm × 5 cm of CU, NM, and CN&CP were randomly cut from the upper, middle, and lower layers of each cage. A brush was used to scrape the surface substrates into a Petri dish, where they were fixed with 4% formalin and 1.5% Luger’s solution and then made up to 50 mL and stored in a 100 mL plastic specimen bottle for testing. The 0.1 mL samples were aspirated using a pipette and placed into a 0.1 mL microplankton counting box, where small organisms such as diatoms and larger organisms such as filamentous algae and invertebrates were identified and counted under an Olympus FX380 microscope (Olympus Corporation, Tokyo, Japan).

#### 2.5.4. Determination of Seedling Growth, Total Yield, and Survival Rate

When releasing seedlings, the initial weight of the seedlings in each net cage was measured, with the average weight and total yield of *A. japonicus* at 12 weeks (replacing the net cage) and 17 weeks (harvesting) measured. A total of 30–300 seedlings were randomly selected and placed in a culture dish to measure their body weight. After drying the seawater on the surface of the seedlings, their total weight was measured using an electronic balance, and the following formula was used to calculate their average body weight (Wa). Then, the specific growth rate (SGR) and survival rate (Sr) of the seedlings were also calculated, as follows:

Wa (g) = m/N(1)

m: total weight of seedlings (g); N: quantity of seedlings.

SGR (%/d) = 100 × (lnWt − lnW0)/t(2)

W0: initial weight (g); Wt: weight of seedlings at time t (g); t: experimental duration (d).

Sr (%) = 100 × Nf/Ni(3)

Nf: final quantity; Ni: initial quantity.

### 2.6. Data Analysis

SPSS v26.0 software (IBM, Armonk, NY, USA) was used to analyze the data, and all data are expressed as means ± standard deviations (SDs). The Kolmogorov–Smirnov method was used for normal distribution detection, and one-way ANOVA (analysis of variance) and Tukey’s HSD (honestly significant difference) were used for comparative analysis of differences. *p* < 0.05 was considered significant.

## 3. Results

### 3.1. Composition of Attached Biological Species on Different Substrates Surfaces

A total of 7 phyla, 23 genera, and 31 species of attached organisms were identified on the surfaces of various substrates in the seedling net cages, including mostly Bacillariophyta (19) and Chlorophyta (4) (Table 1). Furthermore, it also includes two species each for Pyrrophyta, Chrysophyta, and Chordata, as well as one species each for Cyanophyta and Annelida. A total of 27 species were identified on the NM and CN substrate, accounting for 87% of all species. The CP had two more species of algae than the CN, namely *Navicula membranacea* and *Pseudo*-*Nitzschia sicula*. The numbers of alga species attached to the CU were the highest, reaching 31, accounting for 100%.

### 3.2. Variations in the Diatom Density on Different Substrates

**Figure 4:**
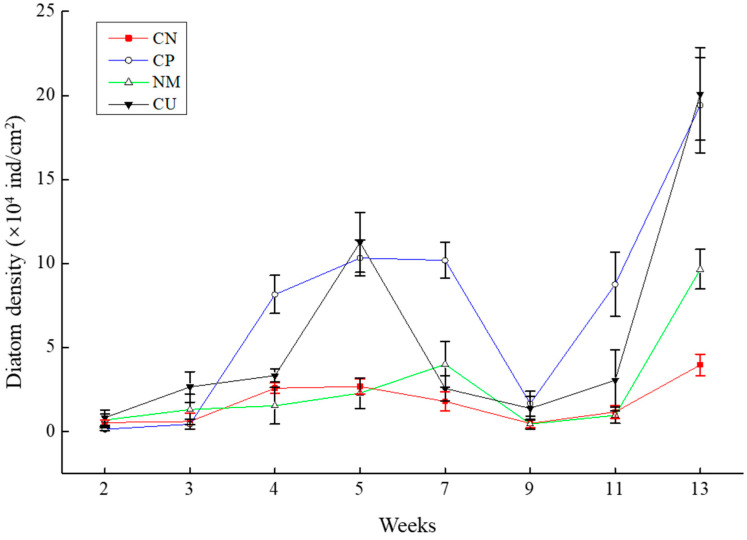
Variations in the diatom density on different substrates CU, CP, NM, and CN. Data are expressed as means ± SD (*n* = 3).

The density of diatoms attached to the substrates generally shows an increasing–decreasing–increasing trend (Figure 4). In week 9, the diatom density almost dropped to its lowest value, and, in week 13, the substrate surface diatom density was in the order CU > CP > NM > CN.

### 3.3. The Percentage of Dominant and Sub-Dominant Species

**Figure 5:**
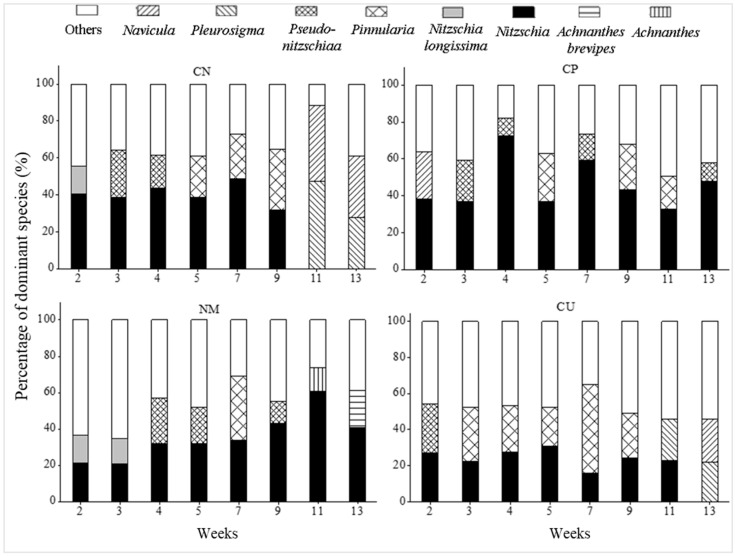
Variations in the dominant species on different substrates.

The percentages of the dominant and sub-dominant species in the substrate surface diatom communities are shown in Figure 5. In week 9, *Nitzschia* sp. were dominant; however, in week 11, *Pleurosigma* sp. was the dominant species, accounting for 47.14%. of identified organisms, and, in week 13, *Navicula* sp. was dominant, accounting for 33.38%. The majority of the CP was dominated by *Nitzschia* sp., with a proportion of 72.66% in week 4, while, in the NM in week 5, the dominant species was rhombic algae. In week 7, *Pinnularia* sp. dominates, and, from week 9 to 13, *Nitzschia* sp. is once again the dominant species, this time with a higher proportion. On the CU, the dominant species in week 2 was *Nitzschia* sp., changing to *Pinnularia* sp. in week 3 before returning to *Nitzschia* sp. in weeks 4–5 and then back to *Pinnularia* sp. in week 7. The *Nitzschia* sp. occupies the dominant species again in weeks 9–11, as well as in week 13.

### 3.4. The Changes in the Total Number of Algae on the Four Substrates

**Figure 6:**
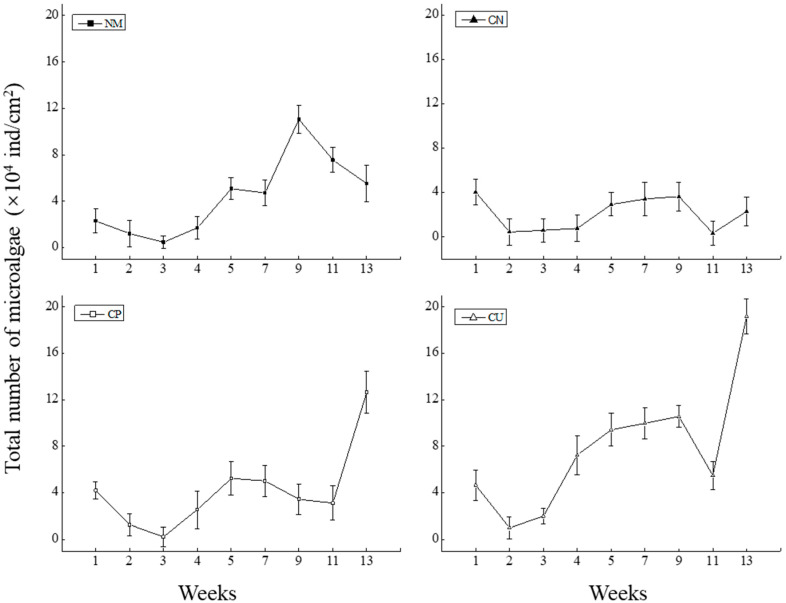
Variation in the total numbers of microalgae on substrates of different materials: NM, CN, CP, and CU.

The changes in the total number of algae on the four substrates all showed a trend of first decreasing and then increasing (Figure 6). The total numbers of algae on the NM, CN, and CP substrates all showed a downward trend in the first three weeks, while the numbers on the NM, CN, and CU increased significantly from weeks 3 to 9. In week 11, the total number of algae on all four substrates decreased, and, in week 13, the number on the CU was significantly higher than the other three substrates (*p* < 0.05).

### 3.5. Changes in DW and AFDW on the Surface Periphyton of Different Substrates

**Figure 7:**
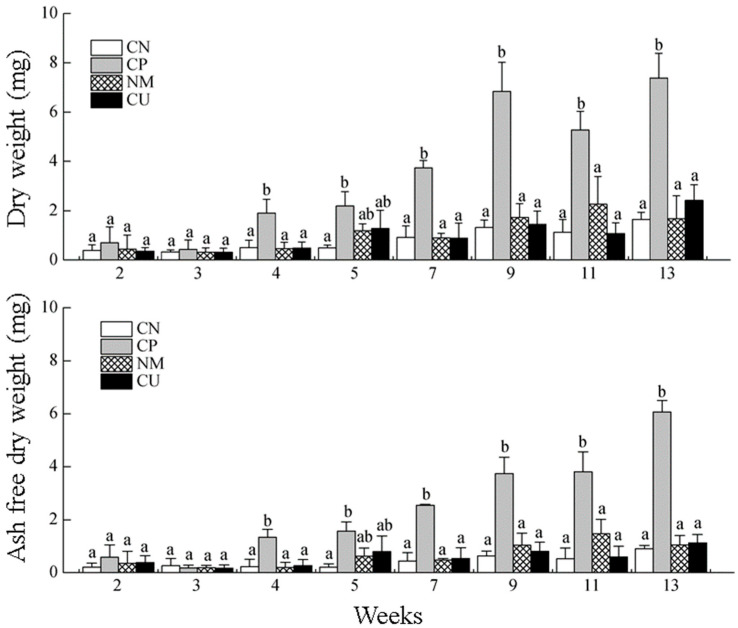
Variations in DW and AFDW of the periphyton. Data are expressed as means ± SD (n = 3); the bars with different letters indicate significant differences (*p* < 0.05).

The DW and AFDW on the surface of the CP substrate increased significantly after week 1 (*p* < 0.05), while the other three substrates showed relatively slow changes (Figure 7). The DW and AFDW of attached organisms on the CN, CP, and CU showed slow changes in week 3, while they showed an increased trend on the CP between weeks 1 and 9. During the entire period, the DW and AFDW on the CN, NM, and CU increased slowly. The DW on the CP decreased in week 13, while the AFDW decreased in week 11. There was no significant difference (*p* > 0.05) in DW or AFDW on the CN, NM, and CU, while, at week 13, the DW and AFDW of the NM, CU, and CP were significantly higher than those of the CN (*p* < 0.05).

### 3.6. Changes in Chlorophyll-a and Pheophytin-a Contents of Algae Attached to Surfaces of Different Materials

**Figure 8:**
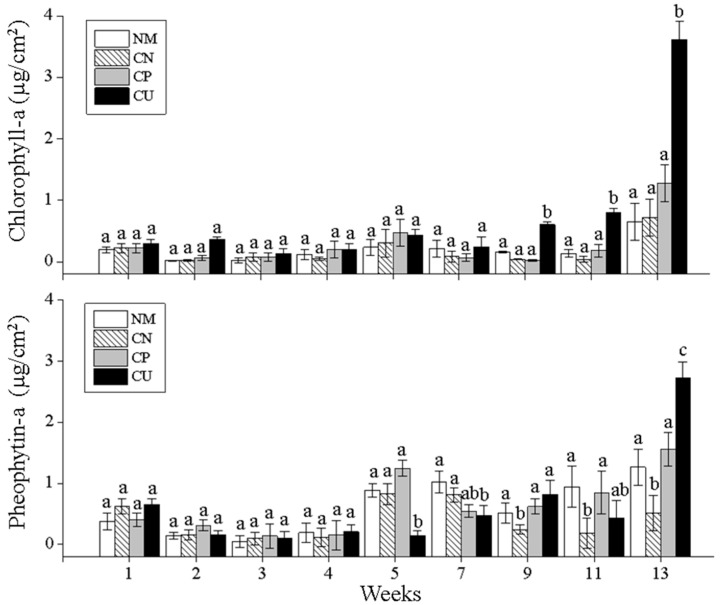
Variations in chlorophyll-a and pheophytin-a of the periphyton on different substrates. Data are expressed as means ± SD; the bars with different letters indicate significant differences (*p* < 0.05).

During the experiment, the chlorophyll-a content of algae attached to the surfaces of the four materials showed a decreasing–increasing–decreasing trend (Figure 8). After week 9, the chlorophyll-a content of the CU was significantly higher than that of the CN, NM, and CP (*p* < 0.05), and the chlorophyll-a content of all the substrates reached its highest values in week 13. The pheophytin-a content showed a decrease–increase–decrease trend, with a significant increase at week 5 (*p* < 0.05), at which point the pheophytin-a contents on the CN, CP, and NM were significantly higher than on the CU (*p* < 0.05). With the decrease in chlorophyll-a content, the pheophytin-a on the CP increased significantly.

### 3.7. Changes in Growth, Yield, and Survival Rate of Seedlings

After week 12, the average weight of the seedlings attached to the CU was the highest, followed by the CN, CP, and NM, with no significant difference among them (*p* > 0.05) (Table 2). The seedling yield on the CU reached 0.92 kg/m^3^, which was 3.68 times larger than the initial weight and significantly higher than that of the CN and CP (*p* < 0.05). The survival rate of seedlings on the CU was the highest, reaching 33.9%, significantly higher than that of the CN, CP, and NM (*p* < 0.05), and the CU also had the highest growth rate, followed by NM, with the CN/CP having the smallest, with no significant difference between them (*p* > 0.05).

After week 17, the average seedling weight, yield, survival rate, and specific growth rate of seedlings showed significant changes, with those on the CU being significantly higher than those on the NM (*p* < 0.05); however, there was no significant difference between the NM, CN, and CP values (*p* > 0.05). The CU yield was significantly higher than that of the others (*p* < 0.05).

## 4. Discussion

### 4.1. Analysis of the Occurrence and Succession of Algae on the Surface of Substrates

To date, many studies have reported the use of artificial substrates for algae and other attached organisms [22,23]. The natural attachment bases vary in shape, size, and surface structure, and these differences make it difficult for quantitative research on attachment organisms [24,25]. Artificial substrates reduce the complexity of natural conditions due to their determined material, size, and shape [25]. The substrate materials selected in this study are commonly used in production, with different functions and convenient sampling. Nylon mesh is the most widely used material in indoor seedling cultivation, ground cages are the most commonly used in sea cucumber pond cultivation and scallop aquaculture, and curvimurate mesh is a polyethylene material used in recent years for seedling preservation production.

The accumulation and formation of attached organisms on substrate surfaces are the result of a comprehensive process of biological migration, reproduction, death, and feeding [26,27]. In this study, a total of 31 species of benthic algae belonging to 23 genera and 7 phyla were identified on attachment substrates made of different materials. Among them, 19 species of Bacillariophyta, accounting for 61% of identified organisms, were in a dominant position, alongside Chlorophyta 4, accounting for 13%, and 2 species each of Pyrrophyta, Chrysophyta, and Chordata, as well as 1 species each of Cyanophyta and Annelida. This may be related to the cell structure and living habits of benthic diatoms, especially certain species that have viscous structures, such as glial stalks and tubes formed by extracellular polymeric substances, which give them a strong adhesion ability.

### 4.2. Changes in Dry Weight and Ash-Free Dry Weight on the Surface of the Substrates

Over the course of this study, the dry weight (DW) and ash-free dry weight (AFDW) of the CP surface increased significantly, which may be related to the stronger water absorption capacity of the CP compared to the other materials. Due to its small mesh size, the number of algae attached to the CN was relatively small, and it is not suitable for the growth of other attached organisms. The amount of organic particles and other pond sediments attached is also relatively small, resulting in a low surface DW and AFDW. The CU has a 14 mesh with a certain permeability configured in a flat state in the net cage, which means that it may uniformly receive light. As a result, alga cells can grow rapidly and effectively absorb pond sediments. This may be the main reason for the stable and rapid growth of DW on the CU. In the experiment, the NM was hung in a net cage with nylon ropes strung together; the mesh unfolds in the water and was located in the different pond water layers, allowing organisms to attach to the mesh in these different layers. Moreover, its mesh size was large, which could better accommodate water sediment. This may be the main reason for the stable DW growth of NM. It has been reported that organic debris is an important food source for detrital benthic communities in marine ecosystems [28], and the quantity and quality of sediment particles play a crucial role in the distribution and metabolism of benthic communities [29,30,31].

### 4.3. Changes in Chlorophyll-a and Pheophytin-a Contents on the Surface of Attached Substrates

The accumulation process of chlorophyll-a on the surfaces of attached substrates may be regarded as the growth process of algae, showing different advantageous conditions for alga growth under different attached substrates. However, the content of chlorophyll-a is not only affected by the number of algae on attached substrates, but also closely related to the type of algae.

The chlorophyll-a content on the NM, CN, and CP showed a similar accumulation process, with a low content and slow growth rate. The reason may be that the NM has uneven lighting and a slow algal growth. The CN’s mesh size was too large, and the effective attachment area of algae was small, which is not suitable for the attachment and growth of algae. Furthermore, other organisms on the CP, such as lime worms, may increase competition for the growth space of algae. Although the chlorophyll-a content on the CU showed no significant difference compared to the other three substrates, it exhibited a slow but stable growth process, especially accelerating in week 9 and reaching its maximum value in week 13, consistent with the trend of the total number of attached algae. By the end of September, when the week 11 measurements were taken, the pond had already passed the high-temperature period, and most of the large algae had declined. The water temperature had decreased, and benthic algae, especially rhombic algae, had proliferated in large numbers. Therefore, chlorophyll-a showed a sharp increase.

The pheophytin-a content on the four substrates showed significant changes, with higher levels of pheophytin than chlorophyll-a at weeks 5, 7, and 9. It has been reported that higher pheophytin levels indicate a rapid algal cell growth, proliferation, and metabolism [32]. Studies have shown that, after the death of phytoplankton cells, chlorophyll immediately dissociates [33]. Free chlorophyll is very unstable and sensitive to light and heat, and, under acidic conditions, the magnesium ion in the center of the chlorophyll molecule porphyrin ring is replaced by two hydrogen ions and converted into magnesium-free chlorophyll, which represents the lifeless chlorophyll content in the water.

### 4.4. The Effect of Substrates on the Sea Cucumber Seedling Growth and Survival Rate

Many studies have shown that the use of artificial substrates in ponds can increase animal growth, survival rates, and yields. In *Macrobrachium rosenbergii* aquaculture ponds, the average body weight, daily pond yield, and total yield were significantly higher when using plastic sheet substrates than under control conditions [28]. Moreover, the type of substrate also affects organism growth and survival rates, such as with freshwater crayfish *Cherax quadricarinatus* [34] and western rock lobster *Panulirus cygnus* [35]. However, relatively little research on the effects of substrates on sea cucumber growth and survival has been conducted, with most relevant studies focusing on occurrence probability [31].

In this study, after 12 weeks, the net cage with the CU had the highest yield and survival rate, reaching 33.9%, consistent with the previous study [36]. In week 17, the yield of seedlings using the CU substrate was significantly higher than that of the other substrates (*p* < 0.05), and the survival rate was significantly higher than that of the NM substrate (*p* < 0.05). After week 12, the average seedling weight was 1.050 ± 0.265 g/ind on the CU, and the yield increased by 3.68 times, with a survival rate of 33.9%. By week 17, the average seedling weight had almost doubled, and the yield increased by 5.76 times, with only a slight decrease in the survival rate, which may be related to the faster reproduction of sea squirts and glass sea squirts. Therefore, cultivating sea cucumbers for 17 weeks may result in higher yields of large-sized seedlings. In this study, the CU substrate showed a wavy shape in the water, with better elasticity than the other substrates, meaning that it could easily uniformly receive sediment and had a good permeability in the water [37]. Furthermore, the DW and ash-free dry weight (AFDW) on the CP increased dramatically after week one. During week 5, the fouling organism *Hydroides ezoensis* appeared on the CP. The rapid early growth of DW/AFDW on CP is most likely due to biofouling by *H. ezoensis*, which is detrimental to sea cucumber seedling growth. The study showed that the benthic diatoms grew vigorously, and their abundant surface algae and organic matter could be used as natural feed for the seedlings [38]. In addition, harvesting sea cucumber from the CU substrate is simple and time- and labor-effective, which can reduce production costs.

## 5. Conclusions

The biological communities attached to the surfaces of the different types of substrates had similar succession processes. In the early stage, benthic diatoms dominate, while, in the later stages, blue algae and green algae dominate. However, the CU substrate presents the most alga species at the highest density and the highest chlorophyll-a content. The net cage with the CU substrate also had a significantly higher seedling yield and survival rate than the other substrates; therefore, among the different substrates investigated, the CU substrate is the best choice for use in sea cucumber seedling aquaculture.

This study provides theoretical support for efficient seedling protection in the sea cucumber industry. However, the study focused on a comparative experiment conducted outdoors for sea cucumber aquaculture. Future study should be carried out to investigate the effects of different types of substrates on the succession of periphyton under indoor environmental conditions in this species.

## Figures and Tables

**Table 1:** Species recorded in periphyton on different substrates in net cage for sea cucumber seedling culture.

Phylum	Genera	Species	Attachment Type			
			NM	CN	CP	CU
Bacillariophyta	Achnanthes	*Achnanthaceae* sp.	+	+	+	+
		*Achnanthes brevipes*	+	+	+	+
	Pinnularia	*Pinnularia* sp.	+	+	+	+
	Synedra	*Synedra* sp.	+	+	+	+
	Pleurosigma	*Pleurosigma* sp.	+	+	+	+
	Coscinodiscus	*Coscinodiscus* sp.	+	+	+	+
	Nitzschia	*Nitzschia paradoxa*	+	+	+	+
		*Nitzschia longissima*	+	+	+	+
		*Nitzschia* sp.	+	+	+	+
		*Nitzschia sigma*	+	+	+	+
		*Nitzschia lorenziana*	+	+	+	+
		*Nitzschia closterium*	+	+	+	+
		*Pseudo-Nitzschia sicula*	+	-	+	+
	Fragilaria	*Fragilaria* sp.	-	+	+	+
	Licmophora	*Licmophora abbreviata*	-	+	+	+
	Navicula	*Navicula* sp.	+	+	+	+
		*Navicula membranacea*	+	-	+	+
	Cocconeis	*Cocconeis* sp.	+	+	+	+
	Diploneis	*Diploneis* sp.	+	+	+	+
Pyrrophyta	Peridinium	*Peridinium* sp.	-	-	-	+
	Alexandrium	*Alexandrium* sp.	-	-	-	+
Cyanophyta	Dactylococcopsis	*Dactylococcopsis acicularis*	+	+	+	+
Chlorophyta	Chlorella	*Chlorella* sp.	+	+	+	+
	Cladophorales	*Cladophorales* sp.	+	+	+	+
	Ulothrix	*Ulothrix* sp.	+	+	+	+
	Ulva	*Ulva* sp.	+	+	+	+
Chrysophyta	Chromulina	*Chromulina* sp.	+	+	+	+
	Isochrysis	*Isochrysis* sp.	+	+	+	+
Chordata	Styela	*Styela clavd*	+	+	+	+
	Ciona	*Ciona intestinalis*	+	+	+	+
Annelida	Hydroides	*Hydroides ezoensis*	+	+	+	+

Note: “+” means the species was detected, and “-” means the species was not detected.

**Table 2:** Growth and survival rate of sea cucumber using different substrates after 12 and 17 weeks. Data are expressed as means ± SD; the different lowercase letters in weeks 12 and 17 indicate significant differences (*p* < 0.05).

	Substrates	Weight (g/ind)	Specific Growth Rate (%/d)	Yield (kg/m^3^)	Survival Rate (%)
After 12 weeks	CN, CP	0.933 ± 0.141 ^a^	2.735 ± 0.187 ^a^	0.59 ± 0.02 ^a^	23.8 ± 3.8 ^a^
	NM	0.868 ± 0.172 ^a^	2.857 ± 0.325 ^a^	0.78 ± 0.24 ^ab^	22.9 ± 4.1 ^a^
	CU	1.050 ± 0.265 ^a^	3.107 ± 0.160 ^a^	0.92 ± 0.16 ^b^	33.9 ± 3.6 ^b^
After 17 weeks	CN, CP	1.747 ± 0.190 ^ab^	2.615 ± 0.100 ^ab^	1.03 ± 0.13 ^a^	21.9 ± 3.1 ^ab^
	NM	1.591 ± 0.200 ^a^	2.531 ± 0.109 ^a^	0.93 ± 0.17 ^a^	21.7 ± 2.2 ^a^
	CU	2.037 ± 0.151 ^b^	2.754 ± 0.066 ^b^	1.44 ± 0.23 ^b^	26.6 ± 1.0 ^b^

## Data Availability

The data that support the findings of this study are available from the corresponding author upon reasonable request.

## Abbreviations

The following abbreviations are used in this manuscript:

CU	Curvimurate net
NM	Nylon mesh
CN	Ground cage net
CP	Ground cage plate
DW	Dry weight
AFDW	Ash-free dry weight
